# Facilitators and barriers to the delivery of school-based smoking prevention interventions for children and young people: a protocol for a systematic review of qualitative studies

**DOI:** 10.1186/s13643-018-0715-8

**Published:** 2018-04-06

**Authors:** Fiona Dobbie, Kathryn Angus, Hannah Littlecott, Karen Allum, Valerie Wells, Amanda Amos, Sally Haw, Linda Bauld

**Affiliations:** 1 Institute for Social Marketing, Faculty of Health Sciences and Sport, University of Stirling, Stirling, FK9 4LA UK; 2 DECIPHer (Centre for the Development and Evaluation of Complex Public Health Interventions), Cardiff University, 1-3, Museum Place, Cardiff, CF10 3AT UK; 3 University of Stirling, Stirling, FK9 4LA UK; 4 Usher Institute of Population Health Sciences and Informatics, University of Edinburgh, Edinburgh, EH8 9AG UK; 5 Faculty of Health Sciences and Sport, University of Stirling, Stirling, FK9 4LA UK

**Keywords:** Children, Young people, Health promotion, Humans, Qualitative research, Schools, Smoking

## Abstract

**Background:**

Despite a decline in child and adult smoking prevalence, young people who smoke (even occasionally) can rapidly become addicted to nicotine, with most adult smokers initiating smoking before they are 18. Schools have long been a popular setting to deliver youth smoking prevention interventions, but evidence of the effectiveness of school-based prevention programmes is mixed, and outcomes vary by the type of programme delivered. Existing systematic reviews that explore the factors contributing to the success or failure of school-based smoking prevention programmes often exclude qualitative studies, due to a focus on intervention effectiveness which qualitative research cannot answer. Instead, qualitative research is focussed on the experiences and perceptions of those involved in the programmes. This systematic review will address this gap by updating a 2009 review to examine qualitative studies. The aim is to generate deeper insight to help target resources which have the potential to save lives by preventing smoking initiation among children and young people.

**Methods:**

This systematic review will be searching the following databases: the Cochrane Library, MEDLINE, EMBASE, PsycINFO, HMIC, ERIC, ASSIA, Web of Science and CINAHL. In order to identify additional references, we will consult the reference lists of a sample of systematic reviews and search relevant organizational websites in order to identify appropriate grey literature. The search strategy will include key words and database-specific subject headings relating to smoking, children and young people, health promotion and school. Authors will independently screen, assess data quality and extract data for synthesis. Study findings will be synthesised thematically using ‘best-fit framework syntheses’. This allows for an existing set of themes to be used as a starting point to map or code included studies. These themes are then adapted as coding takes place to accommodate new emerging themes.

**Discussion:**

This review will focus on qualitative studies that seek to examine the barriers and facilitators to the delivery of school-based smoking prevention programmes in order to inform the design of future theory-based interventions in schools to prevent children and young people from smoking.

**Systematic review registration:**

PROSPERO CRD42014015483

**Electronic supplementary material:**

The online version of this article (10.1186/s13643-018-0715-8) contains supplementary material, which is available to authorized users.

## Background

In the last two decades, much has been achieved to reduce smoking prevalence in both the child and adult population through tobacco control policies, particularly in high-income countries such as the UK. Findings from a Scottish school survey of adolescents aged 13–15 years called SALSUS (Scottish Schools Adolescent Lifestyle and Substance Use Survey) tracked a dramatic decline in adolescent’s smoking over 20 years; 30% of boys and girls aged 15 were smoking regularly (defined as smoking at least one cigarette per week) in 1996 compared to just 7% in 2015 [1].

However, despite the recent decline in overall smoking prevalence in the UK [2], Hopkinson et al. [3] estimate that between 2010 and 2011, a total of 207,000 young people in the UK aged 11–15 started smoking. In addition, recent findings (2013/2014) from the Health Behaviour of School-aged Children survey (which provides health and well-being data from young people, aged 11, 13 and 15, and from 43 countries across Europe and North America) found that on average, 22% of boys and 13% of girls who had ever smoked a cigarette did so before the age of 13 or younger [4]. Prevalence of weekly smoking increases with age, with 1% of boys and 1% of girls aged 11 reporting weekly smoking. By the age of 13, this increases to 4 and 3%, and by the age of 15, to 12% and 11%, respectively [5].

Young people can become rapidly dependent on tobacco, and many smokers are addicted before they leave school, nearly 40% before the age of 16 [6]. Smokers who start at an early age tend to smoke more cigarettes per day in adulthood, smoke for longer, are less likely to quit, find it harder to quit and are more likely to die from a smoking-attributable cause [7–10]. Despite the health consequences of smoking generally developing in later life, there is evidence that young smokers can suffer lung function and lung growth impairment and at a greater risk to coughs and shortness of breath [11, 12]. Therefore, youth smoking prevention interventions remain an important public health policy.

Over the last 30 years, schools have been particularly popular settings to deliver youth smoking prevention interventions [13]. The majority of children can be reached through school making this an obvious setting for smoking prevention interventions. However, evidence of the effectiveness of school-based prevention programmes is mixed, and outcomes vary by the type of programme delivered [13]. For example, a review by Flay found the most effective interventions were those that educated young people about social norms and peer influence on smoking behaviour, in comparison with those that just gave information on smoking harm [14]. In addition, previous systematic reviews of school-based smoking prevention programmes have tended to exclude qualitative studies, mainly because their focus is on intervention effectiveness [13, 15] which qualitative research cannot answer. A recent review of smoking interventions for young people used more inclusive criteria, including both qualitative and quantitative studies [16]; however, it had a specific focus on the equity impact of interventions and only included studies reporting smoking-related outcomes for two or more socioeconomic groups. There is, therefore, a gap in the systematic review evidence from qualitative studies [17], which focus on people’s experiences and perceptions. Such studies can highlight important learning and generate deeper insight into the factors that contribute to the success or failure of school-based smoking prevention programmes.

In February 2010, the UK National Institute for Health and Care Excellence (NICE) published guidance on school-based interventions to prevent smoking [18]. As part of the evidence review to create these guidelines, a team of researchers conducted a systematic review of qualitative research published in 1990–2008 [19]. In 2013, NICE published an evidence update [20] which identified new studies and reviews, but did not systematically search for or include recent findings from qualitative studies.

This protocol is for a systematic review to update the existing review [19] with data from any new school-based programmes as well as any further contributions to the evidence for existing programmes (e.g. [21]).

## Review aim and research questions

The aim of the review is to explore the facilitators and barriers to the delivery of school-based interventions to prevent smoking uptake in children and young people. The review will address the following research questions:

1. What factors aid the delivery of effective school-based interventions to prevent the uptake of smoking?
2. What are the barriers to successful delivery of effective school-based interventions to prevent the uptake of smoking?

## Methods

This protocol has been designed using the PRISMA-P guidelines for systematic review protocol development [22] (see Additional file 1).

### Search strategy

Three types of searching will be used. First, to replicate the 2009 review, the following electronic databases will be searched: Cochrane Library, MEDLINE, EMBASE, PsycINFO, HMIC, ERIC, ASSIA, Web of Science and CINAHL. Second, retrospective reference checking of a sample of systematic reviews and articles (e.g. by most recent publication type) will then be conducted by FD and KAn. Finally, website searching (using pre specified search terms) of key organisation and stakeholder groups (see Table 1) will be conducted to identify any unpublished literature. The search strategy (example provided in Table 2) will replicate the search strategy used in the original review with subject headings relating to smoking, children and young people, health promotion and school.

**Table 1:** Websites of key organisations to identify grey literature

• Action on Smoking and Health (ASH) http://www.ash.org.uk/
• ARIF website and database http://www.arif.bham.ac.uk/
• ASH http://www.ash.org.uk/
• ASH Scotland website http://www.ashscotland.org.uk/ash/
• Bandolier http://www.bandolier.org.uk/
• Centre for UK Tobacco and Alcohol Studies http://www.ukctas.ac.uk
• Clinical Evidence http://clinicalevidence.bmj.com/ceweb/conditions/index.jsp
• Cochrane Public Health Group http://www.ph.cochrane.org/en/index.html
• Department for Children Schools and Families http://www.dcsf.gov.uk/index.htm
• Health Scotland http://www.healthscotland.com/
• http://www.nice.org.uk/aboutnice/whoweare/aboutthehda/hdapublications/hda_publications.jsp
• http://www.childrensnsfcasestudies.dh.gov.uk/children/nsfcasestudies.nsf
• NICE public health guidance http://www.nice.org.uk/guidance/index.jsp?action=byType&type=5
• Public Health Observatories’ websites Quit http://www.quit.org.uk
• The Campbell Collaboration http://www.campbellcollaboration.org/
• The Evidence for Policy and Practice Information and Co-ordinating Centre (EPPICentre) Social Science Research Unit Institute of Education, University of London) http://eppi.ioe.ac.uk/cms/
• The Trials Register of Promoting Health Interventions (TRoPHI) http://eppi.ioe.ac.uk/cms/
• TRIP database http://www.tripdatabase.com/index.html
• UK Public Health Association http://www.ukpha.org.uk/

**Table 2:** MEDLINE example full search strategy. This search strategy will be adapted for each database

1. young people.mp. 2. young person$.mp. 3. young adult$.mp. 4. adolescent$.mp. 5. youth$.mp. 6. teenage$.mp. 7. girl$.mp. 8. boy$.mp. 9. exp Adolescent/ 10. Child/ 11. child$.mp. 12. or/1-11 13. exp Schools/ 14. academy.mp. 15. academies.mp. 16. city technology.mp. 17. education centre$.mp.	18. secure unit$.mp. 19. training unit$.mp. 20. secure training.mp. 21. referral unit$.mp. 22. school$.mp. 23. (offender$ adj institute$).mp. 24. further education.mp. 25. or/13-25 26. 25 and 12 27. health promotion.mp. or exp Health Promotion/ 28. health education.mp. or exp Health Education/ 29. primary prevention.mp. or exp Primary Prevention/ 30. (campaign or teach$ or advis$ or counsel$ or promot$ or encourag$).mp.	31. (program$ or lectur$ or train$ or workshop$ or seminar$ or lesson$ or learn$ or curricul$ or course$ or educat$).mp. 32. or/28-31 33. 26 and 32 34. exp Smoking/ or smoking.mp. 35. smok$.mp. 36. tobacco$.mp. 37. cigarette$.mp. 38. nicotine$.mp. 39. ((prevent$ or abstain$ or abstin$ or stop$ or discourag$ or anti$ or no or non) adj2 smok$).mp. 40. or/35-39 41. 34 and 40 42. limit 41 to (english language and yr="2008 - 2017")

### Inclusion/exclusion criteria

The Participants, Interventions, Comparisons and Outcomes (PICO) format [23] has been used to define the search strategy, to which S (setting), T (type of study) and P (type of publication) have been added. We chose PICO over other tools (such as SPIDER and SPICE) because it appeared to be the most relevant and robust tool to identify the type of studies of interest. [24].

### Participants/population

Participants are any child or young person attending primary or secondary school in a country of origin within the OECD (Organisation for Economic Co-operation and Development).

### Intervention(s) and exposure(s)

The intervention will be any type of school-based smoking prevention intervention or programme. This could focus solely on smoking prevention or be included as part of a risk prevention programme (e.g. drugs, alcohol, sexual health). It will be made clear in the results section (of a future publication focusing on findings from the systematic review) which studies are smoking specific and which cover multiple risk factors. Only findings related to tobacco smoking will be included; marijuana smoking and electronic cigarettes will be excluded.

### Comparator(s)/control

Not applicable

### Outcomes

The outcome of this review is to explore the facilitators and barriers to the delivery of school-based interventions to prevent smoking uptake in children and young people.

### Setting

All types of primary and secondary schools (e.g. state, public, special education, young offenders and faith schools) will be included.

### Types of study to be included

Qualitative studies and mixed methods studies with a qualitative component. Pure quantitative studies or RCTs will be excluded (unless they have a process evaluation which includes a qualitative element). Studies will include any of the following qualitative methods: in-depth, semi-structured, open interviews, group discussion, observation and ethnography.

### Publication characteristics

Reference checking of a sample of recent systematic reviews (i.e. by relevant subject area and published within the last 5 years), journal articles and grey literature published in English between 2008 and 2017 will be eligible for review, using pre-specified search terms. Conference abstracts will be excluded due to a lack of data, although a search will be made for a full-text paper.

## Data management

Once the search terms have been piloted and finalised, electronic databases will be searched and references exported to RefWorks (ProQuest LLC) bibliographic software for storage and removal of duplicates. After removing duplicates, title and abstracts will be reviewed to identify relevant studies using a pre-defined checklist based on the inclusion/exclusion criteria described in the previous section. FD will screen all references, with second review shared by three other members of the research team (KAl, HL, KAn). Full papers will be retrieved for studies deemed potentially relevant. Double screening of full papers will be conducted (FD, KAl and KAn), and those deemed irrelevant will be removed. Where two reviewers disagree, a third reviewer will screen the full paper for inclusion or exclusion. This will then generate a final list of studies for full review. At this stage, the other elements of the search method will be conducted (website review and reference checking).

## Quality assessment

All studies (including grey literature) that meet the inclusion criteria for full review will be independently reviewed by FD, KAl and KAn. In line with the previous review, the critical appraisal checklist developed by NICE for qualitative studies will be used to review the selected articles [25]. NICE provides evidence-based national guidance and advice to improve health and social care, predominantly in England but also to the rest of the UK. It is sponsored by the Department of Health. For each qualitative study, the tool will assess 14 items under the following domains: the theoretical approach and clarity of its aims; the rigour of the methods; how well the data collection was carried out; the relationship between the researcher and participants and reliability of the methods; the richness of the data and rigour and reliability of the analysis and findings; and the reporting of ethical issues. An overall assessment of the study’s relevance and one of three final gradings will be given according to how many of the checklist criteria have been fulfilled, and if not fulfilled, whether the conclusions are likely to alter or not. A grading of ‘++’ will be made if all or most of the checklist criteria have been fulfilled, where they have not been fulfilled, the conclusions are very unlikely to alter; ‘+’ if some of the checklist criteria have been fulfilled, where they have not been fulfilled, or not adequately described, the conclusions are unlikely to alter; and ‘–’ if few or no checklist criteria have been fulfilled and the conclusions are likely or very likely to alter [25].

## Strategy for data collection and synthesis

Data will be extracted by FD and KAl using a ‘data extraction template’ (created using Microsoft Word and piloted with two articles first) that will record the following: aims of research, sample, country where research was conducted, research design and key findings (of relevance to the particular aims of this review only, and quality score from critical review). Study findings will be synthesised thematically using best-fit framework synthesis [26]. This allows for an existing set of themes to be used as a starting point to map or code included studies. These themes are then adapted as coding takes place to accommodate new emerging themes. This approach is particularly useful for the proposed systematic review in three ways. First, it enables utilisation of the themes identified in the previous systematic review [19]. Second, it is an efficient and pragmatic approach to coding when timescales are limited. Third, the framework approach involves ‘charting’ of data in a matrix which allows for greater transparency of coding and, thus, supports coding and analysis via a team of researchers [27]. Our synthesis will be mindful of ‘dissonant voices’ and note themes that are discussed from a range of different perspectives. For example, using peer educators to deliver smoking prevention messages may be positive in terms of reach and credibility but could also be problematic if they are not conveying accurate messages. Once data is extracted, it will be examined for similarities and dissonance. It is likely that, initially, findings will be grouped by setting (primary or secondary school) and by population (pupils, teaching staff, other staff, parents). The review will be reported with reference to the ENTREQ (ENhancing TRansparency in the REporting of Qualitative health research) statement [28].

## Discussion

Much has been written about the effectiveness of school-based interventions to prevent children and young people from smoking, with mixed results depending on the type of programme delivered [13, 14, 29, 30]. However, little is known about the factors that influence their effectiveness. This review will focus on qualitative studies that seek to address this gap by examining the barriers and facilitators to the delivery of school-based smoking prevention programmes. This will generate greater insight to inform the design of future theory-based design interventions in schools to prevent children and young people from smoking. This could help target resources appropriately and has the potential to save lives by preventing smoking initiation among children and young people.

There are some limitations to the outlined systematic review. The restriction to English is acknowledged as a language bias. The cost of high-quality translations of in-depth qualitative data are beyond the resources of this review; however, non-English language studies identified at the screening stages and excluded from the synthesis will be listed in an appendix to the review to aid future reviewers.

## Additional file

Additional file 1: PRISMA-P checklist. (DOCX 29 kb)

## Abbreviations

ASSIA: Applied Social Science Index and Abstracts
CINAHL: Cumulative Index to Nursing and Allied Health Literature
ENTREQ: ENhancing Transparency in the REporting of Qualitative health research
ERIC: Education Resources Information Center
HMIC: Health Management Information Consortium
NICE: National Institute for Health and Care Excellence
OECD: Organisation for Economic Co-operation and Development
PICO: Participants, Interventions, Comparisons and Outcomes
PRISMA-P: Preferred Reporting Items for Systematic review and Meta-analysis Protocols
RCTs: Randomised controlled trials
SALSUS: Scottish Schools Adolescent Lifestyle and Substance Use Survey
SPICE: Setting, Perspective, Intervention, Comparison, Evaluation
SPIDER: Sample, Phenomenon of Interest, Design, Evaluation, Research type
